# Porous silicon embedded in a thermoresponsive hydrogel for intranasal delivery of lipophilic drugs to treat rhinosinusitis

**DOI:** 10.1016/j.jconrel.2023.09.045

**Published:** 2023-10-04

**Authors:** Shrishty Bakshi, Preeti Pandey, Yousuf Mohammed, Joanna Wang, Michael J. Sailor, Amirali Popat, Harendra S. Parekh, Tushar Kumeria

**Affiliations:** a School of Pharmacy, The University of Queensland, Queensland 4102, Australia; b Therapeutics Research Group, Diamantina Institute, University of Queensland, Brisbane, Queensland 4102, Australia; c Department of Radiology, School of Medicine, Stanford University, Stanford, CA 94305, United States of America; d Department of Chemistry and Biochemistry, University of California-San Diego, La Jolla, CA 92093, United States of America; e School of Materials Science and Engineering, The University of New South Wales, New South Wales 2052, Australia; f Australian Centre for Nanomedicine, The University of New South Wales, New South Wales 2052, Australia

**Keywords:** Stimuli-responsive hydrogels, Mometasone furoate, Corticosteroid, Human nasal mucosal tissue, Poloxamer 407

## Abstract

Intranasal delivery is the most preferred route of drug administration for treatment of a range of nasal conditions including chronic rhinosinusitis (CRS), caused by an infection and inflammation of the nasal mucosa. However, localised delivery of lipophilic drugs for persistent nasal inflammation is a challenge especially with traditional topical nasal sprays. In this study, a composite thermoresponsive hydrogel is developed and tuned to obtain desired rheological and physiochemical properties suitable for intranasal administration of lipophilic drugs. The composite is comprised of drug-loaded porous silicon (pSi) particles embedded in a poloxamer 407 (P407) hydrogel matrix. Mometasone Furoate (MF), a lipophilic corticosteroid (log P of 4.11), is used as the drug, which is loaded onto pSi particles at a loading capacity of 28 wt%. The MF-loaded pSi particles (MF@pSi) are incorporated into the P407-based thermoresponsive hydrogel (HG) matrix to form the composite hydrogel (MF@pSi-HG) with a final drug content ranging between 0.1 wt% to 0.5 wt%. Rheomechanical studies indicate that the MF@pSi component exerts a minimal impact on gelation temperature or strength of the hydrogel host. The *in-vitro* release of the MF payload from MF@pSi-HG shows a pronounced increase in the amount of drug released over 8 h (4.5 to 21-fold) in comparison to controls consisting of pure MF incorporated in hydrogel (MF@HG), indicating an improvement in kinetic solubility of MF upon loading into pSi. *Ex-vivo* toxicity studies conducted on human nasal mucosal tissue show no adverse effect from exposure to either pure HG or the MF@pSi-HG formulation, even at the highest drug content of 0.5 wt%. Experiments on human nasal mucosal tissue show the MF@pSi-HG formulation deposits a quantity of MF into the tissues within 8 h that is >19 times greater than the MF@HG control (194 ± 7 μg of MF/g of tissue *vs*. <10 μg of MF/g of tissue, respectively).

## 1. Introduction

Rhinosinusitis is one of the most common sinus and nasal inflammatory conditions, affecting approximately 12 to 16% of Americans and 7–10% of Australians and Europeans [1–3]. Chronic rhinosinusitis (CRS) is the most prevalent type of rhinosinusitis condition [4]. CRS patients suffer from inflammation within the nasal cavity caused by blocked mucous drainage and a resulting mucus build-up in the sinuses [5]. Conventional medications for CRS include steroid-based nasal sprays and decongestant drops that reduce sinus swelling and relieve symptoms. Generally, a direct intranasal delivery of anti-inflammatory drugs is advantageous for CRS treatment due to rapid onset of the relief, low-off target effects, and evasion of first-pass metabolism. Whereas surgical intervention to expand the narrowed sinus (*via* functional endoscopic sinus surgery, or FESS) is a preferred approach for more severe cases, this approach is associated with complications [6]. There are several intranasal anti-inflammatory drug sprays on the market for CRS. However, the conventional intranasal drug sprays are rapidly cleared from the nasal cavity through mucociliary clearance and *via* anterior/posterior leakage, such that any relief from inflammation and swelling of the sinuses is short-lived [7]. In addition, the commercial intranasal spray formulations are not able to access the peripheral sinus cavities, thus providing only partial relief [8].

Stimuli-responsive liquid-to-gel converting drug carriers are uniquely positioned to overcome many of the aforementioned challenges. Such drug carrier systems exist in a liquid form before administration, turning into a semi-solid gel upon administration due to stimuli such as changing temperature, pH, ionic composition, or exposure to UV radiation. A broad range of polymeric hydrogels (HGs) with stimuli-responsive features have been reported and thoroughly tested for safety and efficacy [9–11]. A small number of studies have exploited the biocompatibility, stimuli-responsive release, and sustained drug release properties of stimulus-responsive polymer HGs for delivery of therapeutics to treat CRS [12]. Among the stimuli-responsive HGs, thermoresponsive HGs that transition from a liquid-to-gel-like state triggered by changes in ambient temperature are most suitable for intranasal delivery of therapeutics. This is because the room-temperature liquid form of the formulation is readily introduced to the nasal orifices, and the gel that results from the higher temperature inside the nasal cavity is retained for a prolonged period to provide a sustained release [13]. Therefore, in the case of CRS, where patient self-administration and long-term drug retention are key challenges, thermoresponsive HGs are of a particular interest.

As the name suggests, hydrogels are water-based systems and thus incorporation of lipophilic drugs into HGs is a challenging task. HGs containing lipophilic drugs often show drug precipitation and dose-dumping characteristics [14–17]. New materials strategies to formulate lipophilic drugs in HGs are needed, and research efforts along these lines have explored the use of nano-carriers to introduce lipophilic drugs into aqueous HGs. Here the nano-carrier provides a means to load therapeutics (such as biologics or hydrophobic drugs) whose chemical nature is otherwise incompatible with the hydrogel. This allows the delivery of higher quantities of drug, or greater control over the drug’s temporal release profile at the administered site [18]. In recent years, many nano-carriers like liposomes, polymeric nanoparticles, porous inorganic materials, dendrimers, solid lipid nanoparticles, and protein-based systems have been explored to encapsulate these challenging classes of drugs [19–21]. Of these, mesoporous systems offer a distinct advantage for formulating a lipophilic drug due to their ability to maintain the drug in its amorphous state, which enhances aqueous solubility [22–24]. Composite HGs with drug-loaded micro or nanoparticles have been shown to improve drug stability *in-vivo* and to extend residence time of the formulation at the target site [25–27]. In particular, a recent study from Little et al. showed that mometasone furoate (MF), when loaded into poly (lactic-*co*-glycolic acid) (PLGA) microparticles and incorporated into a poly(N-isopropylacrylamide)-based hydrogel, displayed long-term retention and sustained release over four weeks [28]. However, like most polymeric and liposomal drug delivery systems, drug loading in the PLGA particles used in this work was relatively low (4.4 *wt*%), which translates to a need a higher amount of particles to achieve desired drug amount in the hydrogel. Other major challenges with polymeric and liposomal nanoparticle-HG systems are drug precipitation during storage and transport, poor dispersibility, reduced gel strength, and poor biocompatibility [29].

**Fig. 1.**
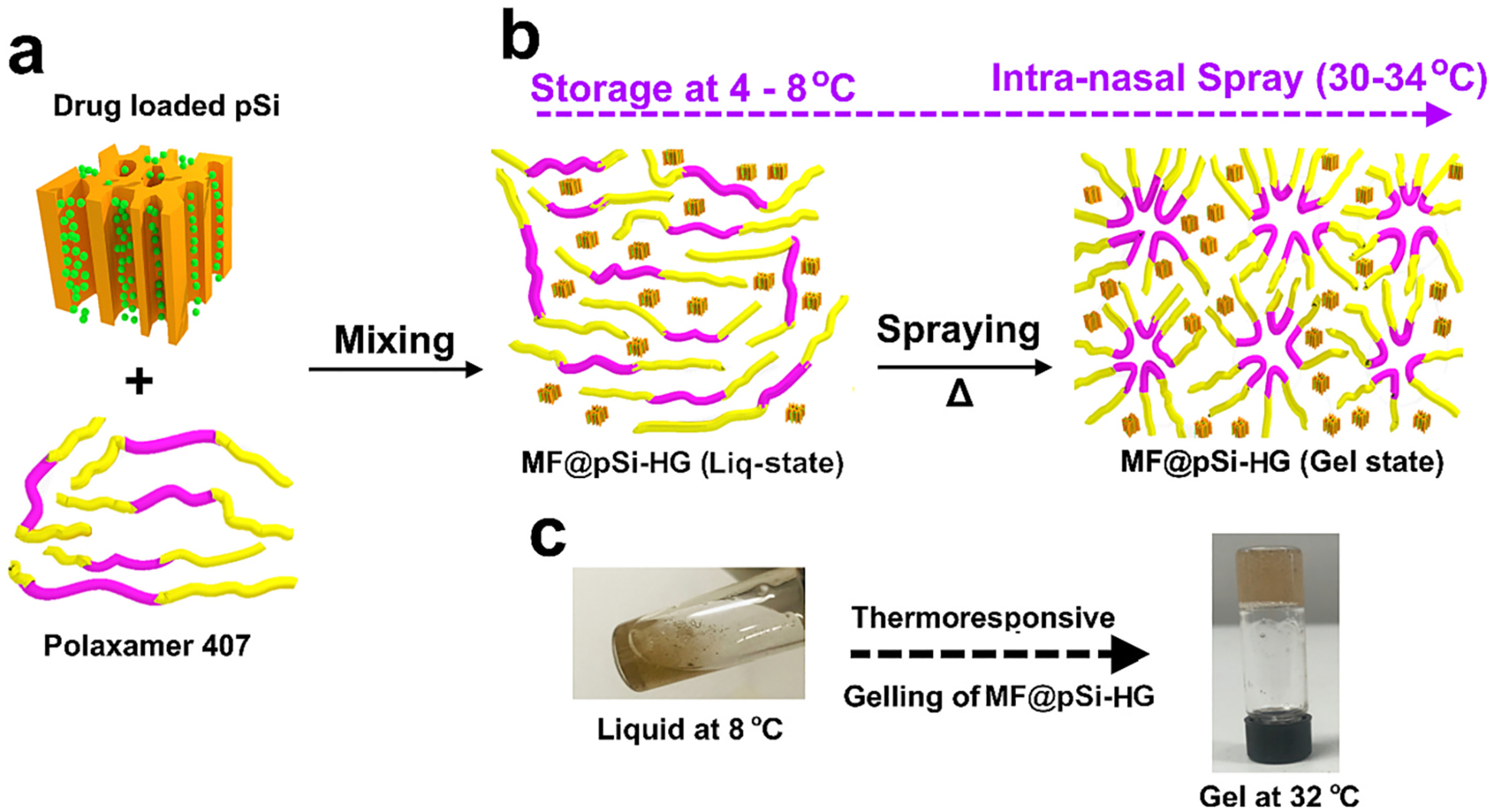
Schematic showing preparation of the composite drug delivery system used in this work, consisting of porous silicon (pSi) particles loaded with the corticosteroid drug mometasone furoate (MF) and dispersed in a thermoresponsive polymeric hydrogel: (a) MF-loaded pSi is mixed with the tri-block polymer poloxamer 407 (P407) to form the MF@pSi-HG formulation. (b) the MF@pSi-HG composite exists as a liquid at storage temperature (4–8 °C), allowing it to be delivered as a spray. The liquid then solidifies to a gel when the temperature rises to a range corresponding to those found in the nasal cavity (30–34 °C). Gelation occurs because P407 transitions into a micellar form. (c) Digital photographs of the MF@pSi-HG system in a liquid state at 8 °C (storage temperature) and viscous-gel state at 32 °C (nasal cavity temperature).

In this study, a lipophilic anti-inflammatory drug (mometasone furoate, MF) was loaded into a porous silicon (pSi) microparticle carrier, which was then incorporated into a thermoresponsive HG. The key properties of the systemrelevant to a controlled intranasal delivery application were then evaluated. Mometasone furoate was chosen as the lipophilic drug due to its proven clinical efficacy and its highly hydrophobic nature. Poloxamer 407 (P407) was chosen as the aqueous thermoresponsive HG host (MF@pSi-HG; Fig. 1). The large surface area of pSi enabled high mass loading of the drug, a degree of control over the temporal drug release profile, and improved solubility, in order to increase bioavailability of the lipophilic drug [30–32]. The composite HG was comprehensively analysed for its physicochemical & rheological characteristics and its ability to deliver MF *in-vitro* in simulated nasal fluid. In addition, safety and drug deposition from the composite HG was evaluated in *ex-vivo* human nasal mucosal explants. Prior works have established pSi-based polymer and lipid composite particle systems as safe (*i.e.,* low cytotoxicity and immunogenicity) carriers with improved *in vivo* drug stability [33–36]. Related composites of pSi carriers with thermoresponsive hydrogels have been used for label-free sensing [37,38]. However, a composite of pSi with a thermoresponsive HG for delivery of lipophilic payloads has not been explored.

## 2. Materials and methods

### 2.1. Materials and reagents

Mometasone furoate (MF) was purchased from Adooq Bioscience (USA, Irvine, CA). Poloxamer (P407), hydroxypropyl methylcellulose (HPMC), glycerine, propylparaben were procured from PCCA (Australia, NSW). SnakeSkin^™^ dialysis membrane (Thermo Scientific, USA), potassium chloride (KCl), calcium chloride (CaCl_2_·2H_2_O), sodium chloride (NaCl), zinc sulphate (ZnSO_4_) was purchased from Sigma-Aldrich, Castle Hill, NSW, Australia. Highly boron-doped p^++^-type crystalline silicon wafers, polished on the (100) face, with resistivity <1.5 mΩ·cm, were purchased from Virginia Semiconductor, Inc. USA. Analytical-grade acetonitrile (ACN) and methanol were obtained from Merck KGa. 24-well culture plates (Corning, NY), cytoTox 96Ò Non-Radioactive Cytotoxicity Assay Kit (Promega, Madison, WI), pen-strep, and Dulbecco’s modified eagle medium (DMEM) (Invitrogen, Carlsbad, CA) were used as-received or following the manufacturer’s instructions.

### 2.2. Synthesis of pSi particles

The pSi particles were fabricated by a combination of mechanical grinding and ultrasonic fracture of electrochemically etched porous silicon film, following a procedure as described previously [39]. First, the silicon wafers were cleaved into smaller pieces and mounted in a Teflon electrochemical etching cell. The silicon was then anodically etched in an aqueous HF-based electrolyte consisting of 3:2 (v:v) 48% aqueous HF: absolute ethanol (*CAUTION: HF is highly toxic and corrosive, and proper care should be exerted to avoid contact with skin, eyes, or lungs*). An etch waveform with current density repeatedly pulsing between a low (50 mA·cm^−2^) and a high (200 mA·cm^−2^) value was used to generate a porous silicon film with high-porosity perforation layer that serves as ab artificial cleavage plane during the ultrasonic milling. The low current density value was sustained for 20 s, and the high current density value had a duration of 1 s. This waveform was repeated through 50 cycles to generate 50 layers of alternating porosities. The electrolyte was replaced with a 3.3% (*v*/v) mixture of 48% aqueous HF in absolute ethanol, and the porous layer was removed from the underlying Si wafer by application of an anodic current pulse (current density of 4.2 mA/cm^2^, applied for 550 s). The freestanding pSi layer was rinsed with copious amounts of ethanol. To obtain pSi particles the free-standing porous Si layers were mechanically ground with an agate mortar and pestle for 2 min, followed by ultrasonication for 20 h (1.9 L Ultrasonic Cleaner, No. 97043, VWR, inc.). For the ultrasonication processing step, the pSi fragments were placed in a 22 mL sealed glass vial containing 10 mL of absolute ethanol (pSi content 2 mg/mL). After ultrasonic fracture, the mixture was allowed to settle for 30 min in the vial, and the supernatant (containing smaller pSi particles) was removed and discarded. The larger, settled particles (average size approximately 900 nm) were used as the drug carriers.

### 2.3. Loading of mometasone furoate into pSi particles

Mometasone furoate (MF) was loaded into the pSi particles by the solvent evaporation method and the mass loading of the drug was fixed at 28 wt% for all pSi formulations in this study. For a typical 50 mg batch of MF@pSi, 14 mg of MF was dissolved in 3 mL of ethanol and 36 mg of pSi particles were added to this solution once the drug was fully dis-solved. This ethanolic suspension of pSi particles with MF was ultra-sonicated for 5 min. This produced a homogeneous suspension of pSi in an ethanolic solution of MF, which was left overnight on an orbital shaker (Thermoline Scientific). The next day, the ethanol was slowly evaporated under vacuum at room temperature for 8 h. This resulted in a dry MF-loaded pSi (MF@pSi) particles powder, which stored at 4 °C until use in preparing composite hydrogel formulations.

### 2.4. Preparation of thermoresponsive hydrogels

The thermoresponsive hydrogel (HG) was prepared following our previously published protocol [40]. Briefly, the pristine hydrogel (*i.e*., no MF or pSi particles) was prepared by dispersing poloxamer P407 (15.5 *wt*%) in a solution consisting of (by mass *i.e*., *wt*%) 0.3% HPMC, 3% glycerol, 0.25% PVA, and 0.02% propylparaben in 5 g of deionized water. The solution was mixed thoroughly for 45 min at 4–8 °C. Afterward, the pH of the mixture was adjusted to pH 5 by addition of small quantities of aqueous HCl or NaOH and the gel was diluted with deionized water to achieve a total mass of 10 g. The final formulation was stored at 4–8 °C until use.

### 2.5. Preparation of thermoresponsive pSi/hydrogel composites

The relevant quantity of MF@pSi particles (Table 1) was dispersed into the pristine hydrogel liquid (see above) to obtain 0.1, 0.2, or 0.5 *wt* % equivalent of MF loaded into the gels. For example, to prepare the 0.1 wt% equivalent MF@pSi-HG 3.6 mg of MF@pSi particles were carefully weighed and added to 1 g of HG and thoroughly mixed to form a homogenous composite hydrogel system. The calculations for required amounts of MF@pSi for the other two formulations (*i.e*. 0.2-MF@pSi-HG and 0.5-MF@pSi-HG) is provided in Table 1. For the preparation of the formulations that consisted of free MF dispersed in HG, denoted MF@HG, the required amount of MF powder was directly added into the liquid form of the HG to obtain 0.1, 0.2, or 0.5 *wt*% equivalent of MF loading. The formulations were vigorously stirred at 4–8 °C for 30 min. As MF does not dissolve in aqueous systems, the final formulations appeared turbid with crystalline MF suspended in the hydrogel.

### 2.6. Physical characterisation of pSi particles

The size of pSi particles was measured using scanning electron microscopy (SEM; Jeol 7001, Japan) and dynamic light scattering (Mastersizer, Malvern, UK). For SEM imaging, the pSi particles were first coated with a 5 nm layer of iridium using a sputter coater (Quorum q150t, Quorum Technologies, UK) to prevent charging, and they were imaged using an accelerating voltage of 10 kV.

### 2.7. Physical characterisation of MF@pSi-HG

Cryo-SEM images were obtained to assess the ultrastructure of the thermoresponsive hydrogel coating on the MF-loaded porous Si-hydrogel composites. A JEOL cryo-SEM was used (JEOL JSM 7100F, Japan). A sample preparation method suitable for hydrated samples was used [41]. The method involved high-pressure freezing (HPF) under vacuum at −210 °C to avoid contamination from atmospheric water and to avoid shrinkage and distortion commonly encountered with conventional dry sample SEM preparation techniques. Measured quantities of the thermoresponsive hydrogel-containing samples were suspended and then frozen. The frozen samples were removed from the specialised HPF caps using a custom-built cap remover, then quickly transferred under vacuum to the cryo-preparation chamber in the frozen state. The frozen samples were fractured *in situ* with a built-in fracture blade and then sputter-coated with Iridium for 120–240 s using a sputter current of 10 mA. The coated samples were moved to the imaging chamber maintained at −145 °C, equipped with an anti-contaminator which was maintained at −194 °C. The samples were sublimed in the chamber at −80 °C for 30 min. The imaging was performed using an accelerating voltage of 7 kV and secondary electron images were collected. The imaging was performed at a working distance of 10 mm.

### 2.8. Rheological measurements of the pSi/hydrogel nanocomposite

Rheological properties of the pSi-HG nanocomposites were measured using a DHR-3 rheometer (TA Instruments, USA) using a 40 mm parallel-plate geometry. The gelation temperature of the nanocomposite gels was determined from oscillation measurements, using a fixed frequency of 1 Hz. First, a stress sweep measurement at 1 Hz (*n* = 1) was performed to determine the linear viscoelastic region (LVR) for further rheological measurements. LVR is the range of shear stress for which the elastic response (G′) of the sample does not change. The shear stress at which G′ deviates from a constant (plateau) value indicates deviation from linear viscoelastic behaviour. After the determination of the LVR, gelation temperature measurement and rheological investigations were performed within this region of shear stress.

For gelation temperature, 0.5 mL of sample was placed onto the plate followed by adjustment the plate-to-cone separation to 200 μm. The samples were allowed to equilibrate for 300 s, at which point the variations in the elastic (G′) and viscous (G″) moduli were recorded as the samples were heated at a rate of 5 °C /min within the temperature range 8–40 °C. Gelation temperature was identified by the transition from a prevalently viscous state (G″ > G′) to one that was prevalently elastic (G′ > G″). All transition temperature measurements were performed in triplicate.

Oscillatory and flow rheometry analysis of the pSi-HG formulations was performed using the above rheometer and accessories. A pSi-HG sample (0.5 mL) was loaded onto the rheometer and allowed to equilibrate for 300 s at the desired starting temperature of the experiment. The plate-to-cone gap was 200 μm for each sample. Values of storage modulus (G′), loss modulus (G″), dynamic viscosity (ŋ’), and loss tangent (tan δ) were obtained from these measurements. The apparent viscosity was measured in temperature ramp mode. To assess the elastic properties of the gels (after formation at the relevant temperature), oscillation tests were performed at a constant temperature (34 °C) and with frequency increasing from 0.1 to 10 Hz. Rheological evaluation tests were performed at 8 °C (cold storage) and 34 °C (typical surface temperature of the nasal mucosa). For each formulation, at least three replicates were acquired, and data collection and calculations were performed using the manufacturer’s TRIOS software [42].

### 2.9. In-vitro release of MF from MF@pSi-HG

Drug release was studied by using the dialysis bag method using a 3.5 kDa molecular weight cut-off (MWCO) SnakeSkin dialysis membrane (Thermo Scientific, USA). Two release media were employed in the drug release studies: simulated nasal fluid (SNF) and SNF diluted with ethanol. The SNF was prepared by dissolving 129 mg of KCl, 745 mg of NaCl, and 32 mg of CaCl_2_ in 100 mL of deionized water. The ethanol diluted SNF mixture contained 50% by volume ethanol with SNF (*i.e*., 50:50 SNF: ethanol by volume). The dialysis tube was filled with 0.5 mL of the MF@pSi-HG formulation, and the sealed assembly was placed in a glass vial containing 7.5 mL of the relevant release medium. The temperature of the chamber was maintained at 34 ± 1 °C. Aliquots (0.5 mL) were collected from the receiver compartment at regular intervals (0 h, 1 h, 2 h, 4 h, 6 h, 8 h) and replaced with an equal volume of fresh release medium after each sampling. The amount of MF released at each time point was quantified using reverse-phase high-pressure liquid chromatography (RP-HPLC). MF release from MF@HG control formulations was determined using the same process as the MF@pSi-HG formulations.

### 2.10. Quantification of MF released from MF@pSi-HG using HPLC

The amount of MF released from MF@pSi-HG or control formulations was analysed using an RP-HPLC system (Shimadzu, SIL-10AXL, Japan) equipped with a low-pressure quaternary gradient pump, a dual-wavelength UV detector, autosampler, C18 column (Phenomenex Luna C18 column of 250 mm length, with an internal diameter of 4.6 mm, and particle size of 5 μm), and column oven (maintained at 25 °C). The chromatographic data were processed using Lab Solution 1.24 SP1 software. For determination of MF in the release aliquots, 10 μL of the sample was injected into the RP-HPLC and a flow rate of 1 mL/min was maintained for 7 min. The mobile phase consisted of acetonitrile and water in a ratio of 85:15 (*v*:*v*). The UV absorbance signature of MF was measured at a wavelength of 248 nm.

### 2.11. Collection and preparation of human nasal tissue

The tissue toxicity and drug deposition from MF@pSi-HG were carried out using freshly excised human nasal mucosal tissue. The tissues were obtained from patients undergoing surgery for nasal obstruction at the Greenslopes Hospital, Brisbane, Australia. The study was approved by the Greenslopes Hospital, and the University of Queensland-School of Pharmacy Human Ethics Committees (HREC/14/QPAH/530), and written consent was obtained from all human subjects donating the nasal tissues. Nasal tissue samples were collected from the middle turbinate, inferior turbinate, or superior nasal septum based on the planned operation and used immediately. The human specimens were carefully dissected with the use of a scalpel from undesired connective tissue and washing for the removal of tissue debris and blood prior to *ex-vivo* testing (toxicity and drug deposition) of the formulations.

### 2.12. Lactate dehydrogenase toxicity assay and MF deposition in human nasal tissue

For assessment of toxicity of the MF@pSi-HG formulations on human nasal mucosal tissue, an organ type (*i.e.,* explant) tissue culture system was first established using the biopsied nasal mucosal tissues and toxicity was measured using lactate dehydrogenase (LDH) assay. Briefly, the human nasal mucosal tissue was carefully cut into 2–4 mm × 2–4 mm × 1–2 mm (L × W × D) cuboidal sections using a scalpel and placed into in 24-well culture plate in such a way that the epithelial side faced upwards and was exposed to air. The tissue section explants in the 24 well plate were maintained viable in 300 μL of culture medium. The culture media consisted of 50% Dulbecco’s Modified Eagle Medium (DMEM) high glucose (Invitrogen, Carlsbad, CA) and supplemented with 25% Hank’s buffered saline solution (Invitrogen, Carlsbad, CA) and 50 U/mL penicillin G and 40 μg/mL streptomycin (Invitrogen, Carlsbad, CA). Next, human nasal mucosal tissue explants were placed in a humidified incubator at 37 °C under 5% CO_2_. The culture media was replaced every 24 h, and tissue explants were incubated for 5 days to obtain stable baseline extracellular LDH readings. After the stabilisation of LDH levels, the tissue explants were exposed to 5 μL of one of the three MF@pSi-HG formulations. SNF (5 μL) was used as a negative control, whereas 1 *wt*% ZnSO_4_ dissolved in DMEM media (5 μL) served as a positive control. The MF@pSi-HG and the negative and positive controls were incubated with the tissue explant for 5 days and LDH levels were recorded every day as per the manufacturer’s protocols. Briefly, a 50 μL aliquot of culture medium was removed from each well, and 50 μL of substrate mix was added. Following 30 min incubation at room temperature, the reaction was stopped with 50 μL of stop solution, and absorbance was read at 490 nm (FLUO star Omega spectrophotometer, BMG Labtech, Offenburg, Germany). The effects of treatments were calculated as fold changes in extracellular LDH levels of tissues. All the toxicity measurements were measured with *n* = 3 tissue explants and the data are presented as mean ± standard deviation.

### 2.13. Histology and imaging of human nasal tissue sections

After 5 days of toxicity screening, the tissue samples were treated with the MF@pSi-HG nanocomposite formulations (0.1-MF@pSi-HG or 0.5-MF@pSi-HG), SNF, and 1 *wt*% ZnSO_4_ were collected for histological evaluation. First, the tissues were fixed using 4% formaldehyde and stored at 4 °C overnight. This was followed by dipping the tissues in 20% sucrose overnight at 4 °C. Afterward, the embedding of specimens in OCT was performed which was followed by freezing at 20 °C and cryo-sectioning in slices. The samples were stained with hematoxylin and eosin (H & E) for 1 min, and the stained tissues were dehydrated in ethanol followed by defatting in xylene. A coverslip was placed over the stained tissue samples for histological evaluation. Tissue integrity was observed *via* histological staining and microscopy to evaluate the safety of the formulation.

### 2.14. MF deposition onto human nasal mucosal tissue

A Franz diffusion setup (Logan instruments Ltd., USA) was used to determine the amount of MF that could be deposited into the tissue from the 0.1-MF@pSi-HG or the control 0.1-MF@HG formulations. The nasal mucosal tissue was packed between donor and receiver compartments of the Franz cell equipped with temperature-controlled water jackets set at 34 ± 1 °C. The nasal tissue was packed such that the dorsal side of the tissue faced the donor compartment, and the ventral side faced the receiver compartment. The donor and receiver compartments were filled with 0.5 mL of formulations and 12 mL SNF, respectively. Following 2 h, 4 h, 6 h, and 8 h of exposure, the tissue samples were collected, rinsed with ultrapure water, dried with lint-free paper, and then wrapped with aluminium foil to protect from light and stored at −80 °C until further analysis.

### 2.15. Tissue sample preparation for HPLC analysis HPLC analysis of MF in human nasal tissue

The tissue samples were thawed to room temperature, blotted dry using filter paper, frozen in liquid nitrogen, ground with a mortar and pestle, and then processed into a homogenate (100 mg/mL) using ultrapure water [43]. The tissue homogenate (10 μL) was mixed with a fixed quantity of methanol to precipitate protein, and an aliquot of 10 μL of the supernatant was injected onto the RP-HPLC-PDA. The instrumentation and chromatographic conditions used for the analysis of MF are given in section 2.10. The assay method was validated using the FDA criteria for bioanalysis from 1 to 100 μg/mL [44].

## 3. Results and discussion

### 3.1. Physical characterisation of the porous silicon-hydrogel composites

**Fig. 2.**
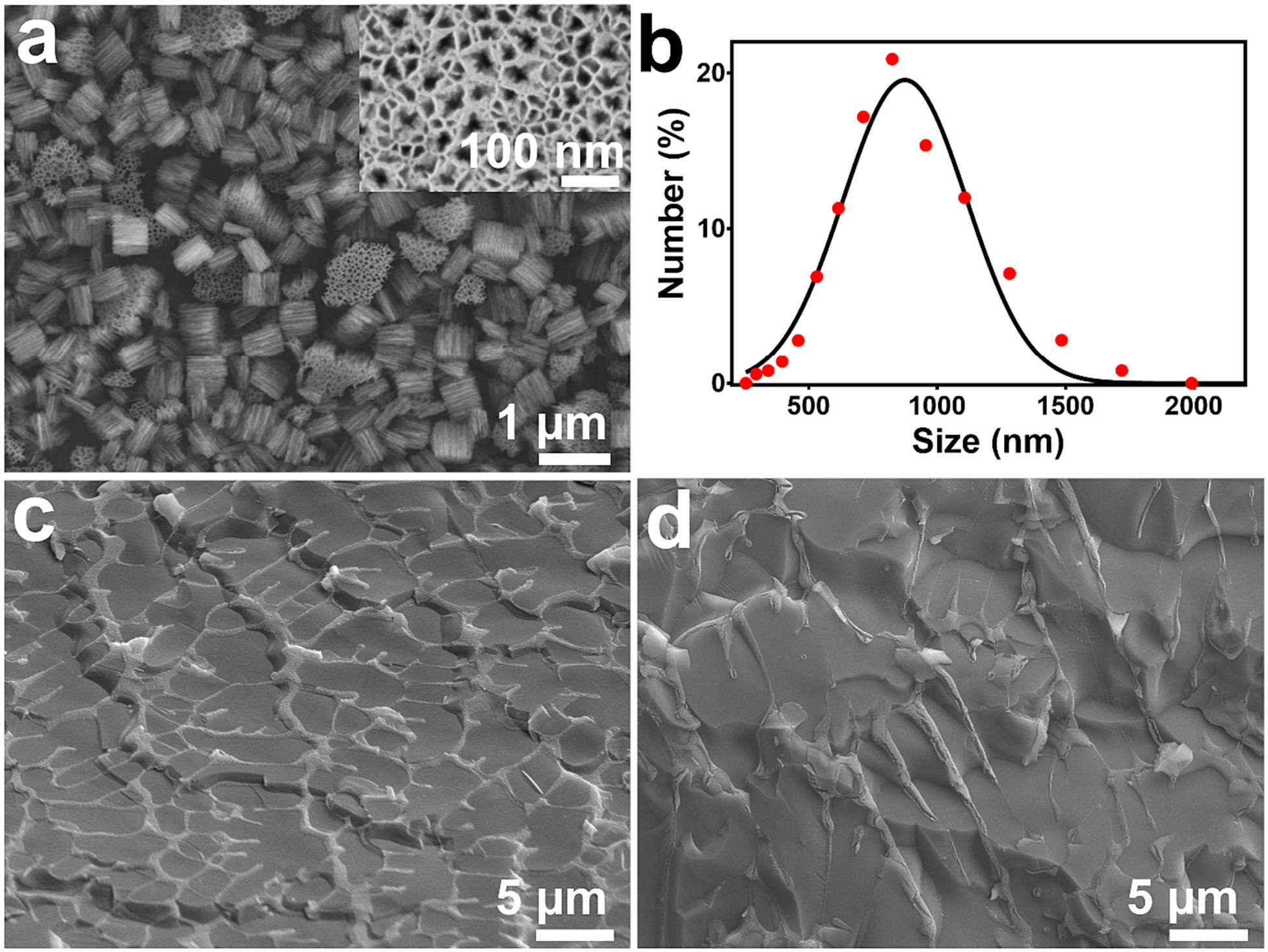
(a) FE-SEM image of pSi particles showing plate-like particles with a characteristic unidirectional pore morphology. A plan-view image of a single particle, showing the pore texture, is shown in the inset. (b) Hydrodynamic diameter (from dynamic light scattering measurement) of representative pSi particles, showing an average particle size of 875 nm. Cryo-SEM images of the hydrogel system prepared using (c) free mometasone furoate (0.5MF@HG), and (d) MF-loaded pSi particles (0.5-MF@pSi-HG).

The approach of the present work involved loading the corticosteroid drug mometasone furoate (MF) into porous Si (pSi) particles, and these particles were then dispersed into a poloxamer 407 (P407)-based thermoresponsive polymeric hydrogel, as outlined in Fig. 1. The rationale for using the nanostructured porous pSi micro particles is to overcome the low solubility of MF in the aqueous media. Free MF forms crystalline aggregates in aqueous solutions, impeding dissolution and negatively impacting tissue bioavailability [45]. The nanostructure of the porous silicon particles is evident in the FE-SEM images (Fig. 2a), which reveal highly directional pores that are characteristic of this type of electrochemically generated porous silicon, and with pore diameters in the range of 10–25 nm. The average hydrodynamic size of the particles was determined by dynamic light scattering to be 875 nm (Fig. 2b), which is consistent with their appearance in the SEM images. The open porosity of the particles, determined using the spectroscopic liquid infiltration method [46] on the films prior to particle formation, was 70%. The high open pore volume and the high porosity are keys to maximize the loading of therapeutic payloads, while the nanoporous morphology maintains the drug in a more bioavailable amorphous form [47]. The MF drug was loaded into the pSi particles using the solvent evaporation method, where the particles were dispersed in a solution of the drug in ethanol and the solvent removed under vacuum. This generally results in drug deposition both within the porous nanostructure and on the surface of the particles. The particles were then formulated with the poloxamer-based thermoresponsive hydrogel, resulting in the system referred to in this work as MF@pSi-HG. As the amount of MF loaded in the pSi particles was fixed at 28% by mass, the amount of MF in a given hydrogel formulation was adjusted by adjusting the mass of MF-loaded particles (MF@pSi) added to a given mass of hydrogel during preparation. Three MF@pSi particle concentrations were compared in this work, denoted 0.1-MF@pSi-HG, 0.2-MF@pSi-HG, and 0.5-MF@pSi-HG, which correspond to mass percentages of MF in the hydrogel of 0.1, 0.2, and 0.5%, respectively (Table 1).

The microstructure of the resulting hydrogel composite was characterised using cryo-SEM measurements. The microstructure of 0.5-MF@pSi-HG and the 0.5 *wt*% MF-loaded hydrogel control formulation (*i.e*., 0.5-MF@HG, containing MF and no pSi) indicated that both the 0.5-MF@HG control (Fig. 2c) and the 0.5-MF@pSi-HG (Fig. 2d) samples exhibit an interconnected polymer network structure. The 0.5-MF@pSi-HG samples contained 13 mg of MF@pSi particles per gram of HG; at this concentration the particles did not appear to influence the microstructure of the HG.

### 3.2. Rheological evaluation of MF@pSi-HG formulations

The gelation temperature of the formulations was measured by analysing changes in the values of storage (G’) and loss modulus (G”). Storage modulus (G’) is a measure of elasticity of the gel state, and the loss modulus (G”) is a measure of the viscous liquid state components of the formulation. The storage modulus of this type of hydrogel increases as the temperature of the material rises through the gelation temperature that is, the viscous liquid material transitions to an elastic gel above a certain temperature. The origin of this unusual behaviour is generally attributed to the interaction between the hydrophobic and the hydrophilic segments of the copolymer units and the aqueous medium in which they reside [48]. Thus, the storage/elastic modulus was lower than loss/viscous modulus (that is, G’ < G”) when the formulation was in its liquid form. As the material underwent the liquid-to-gel transition, the storage/elastic modulus became greater than the loss/viscous modulus (G’ > G”). The temperature of the crossover point, where G’ = G”, is called the gelation temperature or the lower critical solution temperature (LCST).

**Fig. 3.**
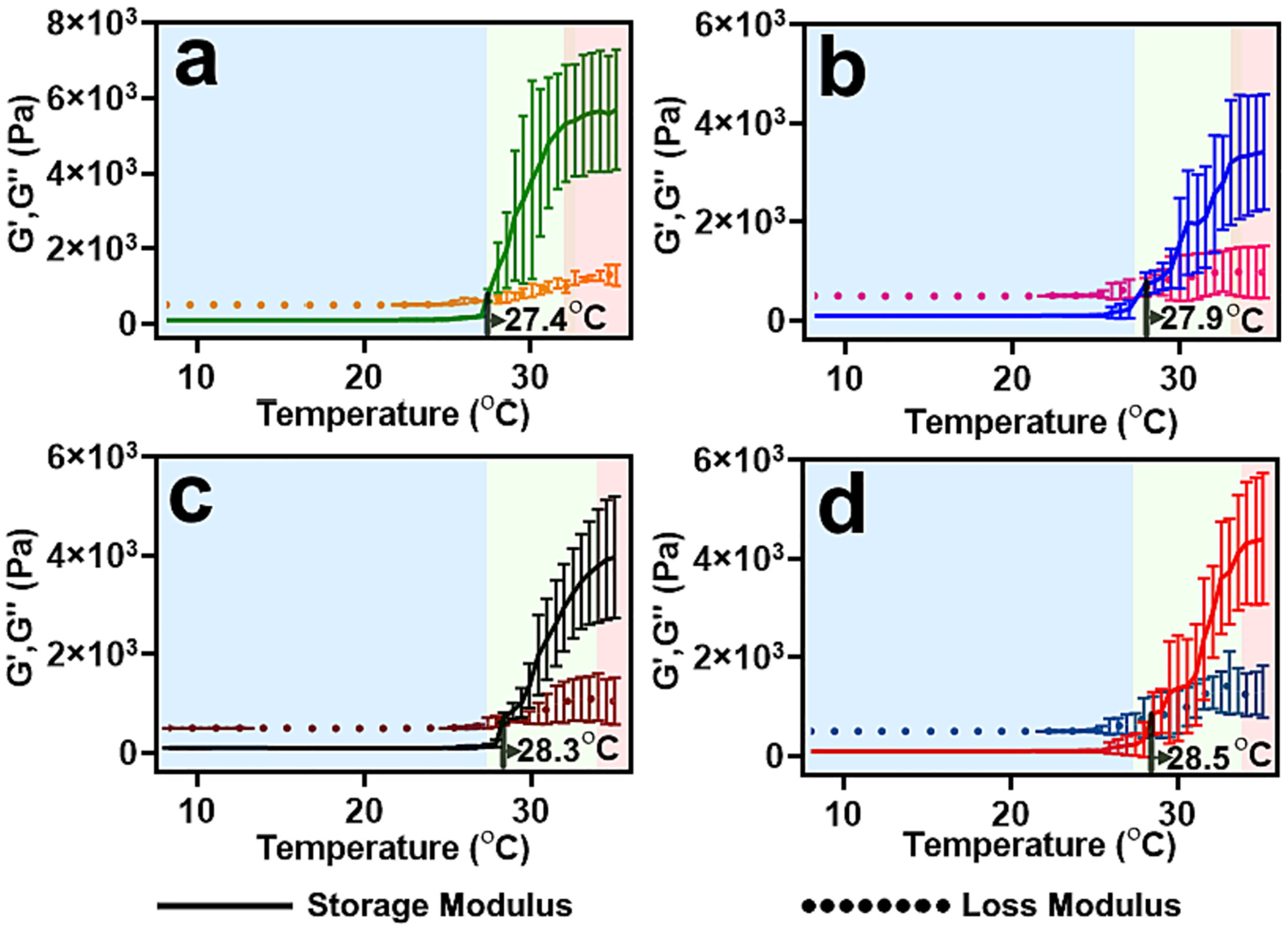
Temperature-dependent changes in storage (G’) and loss (G”) modulus of hydrogel formulations used to determine gelation temperature. The temperature *versus* storage and loss modulus of (a) blank hydrogel, (b) 0.1-MF@pSi-HG, (c) 0.2-MF@pSi-HG, and (d) 0.5-MF@pSi-HG. A sharp rise in the values of storage modulus is observed at the gelation temperature. The different coloured shaded regions in the graphs represent the three gelation stages. The blue coloured region (far left in all plots) corresponds to the liquid phase where the system behaves as a viscous liquid. The green region is the gel phase indicating gelation of the liquid. The red region indicates the formation of a strong and stable gel. The solid line in each graph represents the storage modulus and the loss modulus is presented by a dotted line (*n* = 3 ± SD).

Consistent with the known behaviour of the poloxamer 407 system, the liquid to gel transition profile of these hydrogels consisted of three phases, a liquid phase, a gel phase, and a gel stabilisation phase (Fig. 3). In the first liquid phase (shaded light blue) the values of storage modulus (G^’^) were less than loss modulus (G^”^) characterised by a low viscosity liquid state. In the second gel phase (shaded light green), a drastic increase in the storage modulus (G^’^) occurred, indicating an onset of elastic behaviour. In the later stages of the second phase, the values of storage modulus (G^’^) become substantially greater than the values of loss modulus (G^”^), indicative of stable, elastic, and high-quality gel formation (shaded light red). The rheological data indicate that the formulations that contained pSi particles require longer time for gel stabilisation to occur.

Gelation temperature of the hydrogel composition was adjusted to around 27 °C by systematic adjustment of the concentration of P407 in the formulation, along with other excipients (HPMC, glycerol, PVA, and propylparaben). This gelation temperature was chosen to account for the lower physiological temperature in the nasal cavity (30–34 °C) and to ensure complete gelation would be achieved at nasal cavity/sinus temperatures. The addition of MF-loaded pSi particles exerted only a minor effect on the gelation temperature of the HG formulations, Table S1 (Supporting Information). As shown in Fig. 3 the gelation temperature increased from 27.4 ± 0.003 °C (for the blank hydrogel) to 28.5 ± 0.01 °C for the 0.5-MF@pSi-HG formulation (this latter sample contained 13 mg MF@pSi/g of HG). Overall, the MF@HG and MF@pSi-HG formulations (0.1-MF@pSi-HG, 0.2-MF@pSi-HG, and 0.5-MF@pSi-HG) all displayed a gelation temperature in the targeted range. The minor increase in gelation temperature upon addition of pSi particles is attributed to the hindered entanglement of the P407 chains, which drives the gelling process. While it is established that excipients can impact of properties of hydrogels [49]. In the present work the concentration of pSi particles in the HG was chosen to be sufficiently low that it would exert minimal influence on rheologic performance.

The rheological properties of hydrogel formulations provide key information about their flow behaviour that is important in determining their storage, application, and drug release characteristics. In this regard, the viscosity of the MF@pSi-HG formulations were characterised in the temperature range of 8–37 °C. The viscosity of the formulations increased with temperature for each formulation (Fig. S1, Supporting Information), consistent with the liquid-to-gel state transformation discussed above. Similar to their storage and loss modulus values, the MF@pSi-HG and the blank HG samples showed only minor differences in their viscosity-temperature behaviour.

The tan delta (δ) value is the ratio of loss modulus to the storage modulus. This metric provided a more pronounced measurement of the variations in performance between the different hydrogel formulations (Fig. S2, Supporting Information). The blank HG showed a sharp drop in tan (δ) and an abrupt flattening of the curve at temperatures greater than the gelation temperature (Fig. S2a, Supporting Information). The sharp drop in tan (δ) was also observed for the HG formulations that incorporated MF@pSi particles (Fig. S2b–d, Supporting Information), although the tan (δ) values transitioned to a plateau less abruptly, requiring a substantially larger increase in temperature before reaching a stable value. The temperature range of the transition increased with increasing concentration of pSi particles, suggesting that the pSi particles more effectively hinder entanglement and formation of long-range order in the P407 chains during the gelation process.

The rheological properties of the MF@pSi-HG formulations are summarized in Table S1 (Supporting Information). Overall, the incorporation of MF-loaded pSi particles resulted in a relatively minor shift in the gelation temperature of the hydrogel, and the viscosity and other rheological properties were largely unaffected for the particle concentrations studied in this work.

### 3.3. Dynamic rheological measurements of MF@pSi-HG formulations

The mucus in the general nasal cavity undergoes ciliary movement and coupled with the breathing process, it is a dynamic site with cyclical movement. Therefore, assessment of the viscoelasticity at varying oscillatory frequencies can provide important rheomechanical information about potential stability of a formulation in the nasal environment. The effect of frequency change on viscoelastic properties of HG formulations was studied at two temperatures: the maximum storage temperature (8 °C) and at a temperature slightly greater than the gelation temperature (30 °C). Hydrogel formulations were subjected to sinusoidal stress over a range of frequencies (1–15 Hz) at these temperatures. Higher relative elasticity values imply longer retention at the site of administration and reduction in anterior or posterior leakage of the formulation. The observed higher values of storage modulus (G^’^) over loss modulus (G^”^) indicate the ability of the hydrogel to retain its gel structure throughout the tested frequency range, which confirms the expectation that the poloxamer P407 hydrogel would have a negligible tendency to leak out of the nasal cavity (Fig. S3a–b, Supporting Information). Similar trends of G’ and G”, when measured against frequency changes, were observed for all the HG formulations that contained MF-loaded pSi (Fig. S3c–h, Supporting Information).

For material in the gel state at 30 °C, the storage (G^’^) and loss modulus (G^”^) values of the blank HG and the pSi-HG composite formulations were comparable; all had higher values of storage modulus (G^’^) and lower values of loss modulus (G^”^), albeit there were slight differences in the absolute values of storage modulus (G^’^) in each case. In the case of 0.1-MF@pSi-HG, the values of storage modulus were smaller than those of the blank hydrogel at most of the frequencies studied, which could indicate a reduced interaction and entanglement of the polymer chains in the presence of the pSi particles. By contrast, the 0.2-MF@pSi-HG and 0.5-MF@pSi-HG composite formulations (which contained an increasingly greater quantity of pSi particles), the values of storage modulus (G”) increased with increasing pSi particle content. This could be the result of formation of hydrogen bonds between Si-OH groups present on the pSi particle surface and the hydrophilic regions of the poloxamer chains. Recent studies have reported that the addition of nanoparticles containing surface silanol groups to hydrogels leads to hydrogen bonding with the polymeric chains, resulting in increased storage modulus values [50–52]. This effect was observed when the nanoparticle concentration was above a certain threshold value. Presumably, the number of particles in the 0.1-MF@pSi-HG nanocomposite formulation was below this threshold, while the 0.2-MF@pSi-HG and 0.5-MF@pSi-HG composite formulations were above this threshold.

As expected for the liquid phase, the peak loss and storage modulus values for all the formulations (blank HG and the three MF@pSi-HG samples) measured at 8 °C were significantly lower than those measured at 30 °C. It is worth noting that the peak G’ and G” values for blank HG at 8 °C were almost twice that of the HG formulations that contained MF@pSi particles, despite the fact that the peak G’ and G” values measured at 30 °C were unaffected by the presence of MF-loaded pSi particles. This data suggests that the MF@pSi-HG formulations could be more amenable to spraying into the nasal cavity due to their lower viscoelasticity at storage temperatures while maintaining high integrity at physiologic temperatures.

**Fig. 4.**
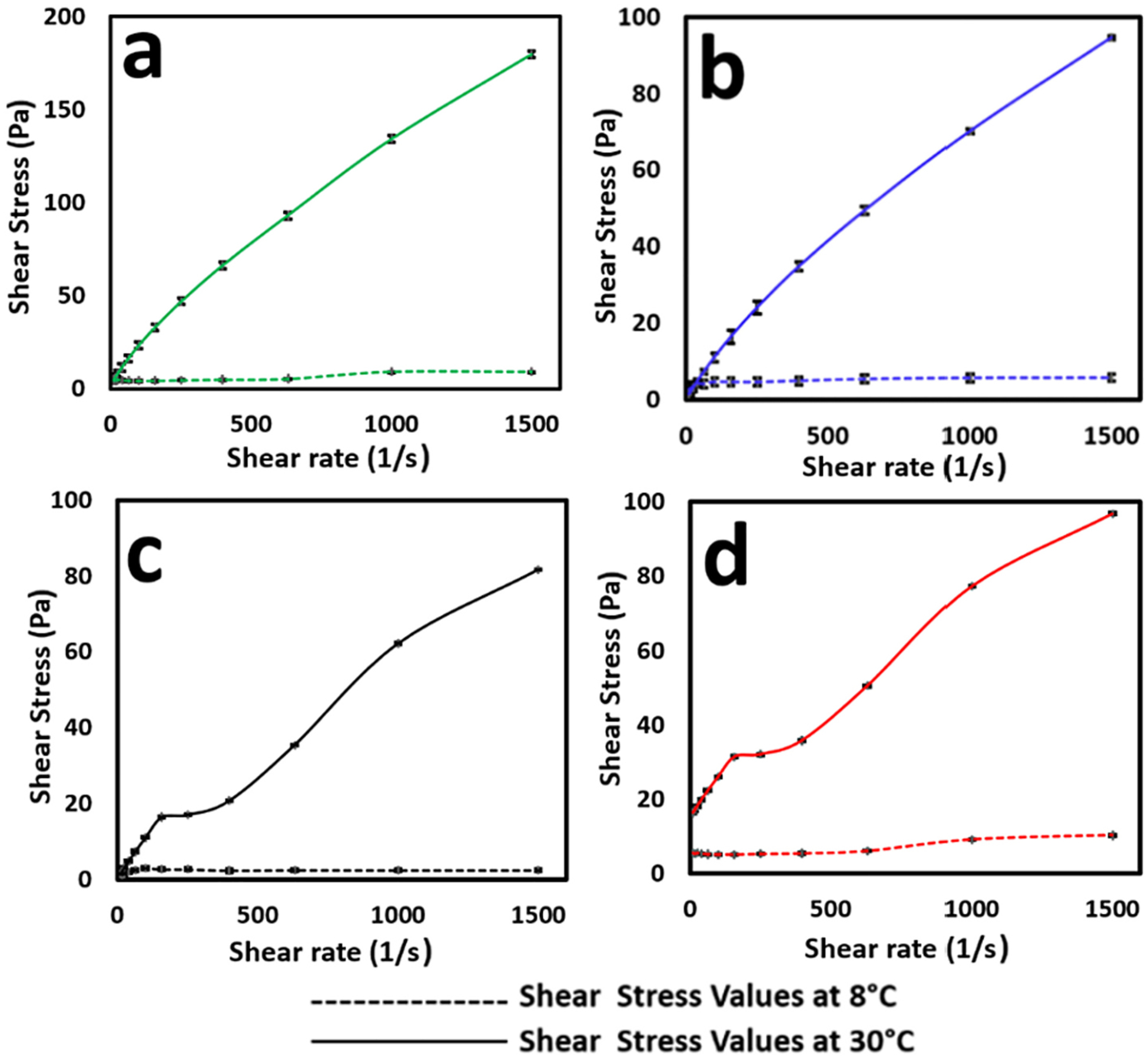
Effect of changing shear rate on shear stress generated in (a) blank hydrogel, and formulations (b) 0.1-MF@pSi-HG, (c) 0.2-MF@pSi-HG, and (d) 0.5-MF@pSi-HG. At 8 °C, when the hydrogel is in its liquid state, changes in shear rate do not result in a significant change in shear stress while at 30 °C (temperature slightly above the gelation temperature), changes in shear rate result in a significant change in shear stress (*n* = 3 ± SD).

The shear stress behaviour of the blank hydrogel and pSi-HG composite formulations (0.1-MF@pSi-HG, 0.2-MF@pSi-HG, and 0.5-MF@pSi-HG), evaluated in their viscous liquid state (at 8 °C), showed minor increases in shear stress when subjected to a range of shear rates (10–1500 per second). In contrast, the shear stress was found to increase significantly near the gelation temperature (30 °C); such an enhanced resistance to flow is consistent with gel formation (Fig. 4). Blank hydrogel (Fig. 4a) formulations displayed higher shear stress than pSi-HG composite (Fig. 4b–d) formulations, indicating that the pSi particles influence the rheological properties of the P407-based gels [53,54]. The reason for the lower values of shear stress measured on the nanocomposite formulations as compared to the blank gel could be due to a marginal reduction in interactions among the polymeric chains in the presence of pSi particles.

### 3.4. Quantification of MF in-vitro release from pSi-HG formulations

**Fig. 5.**
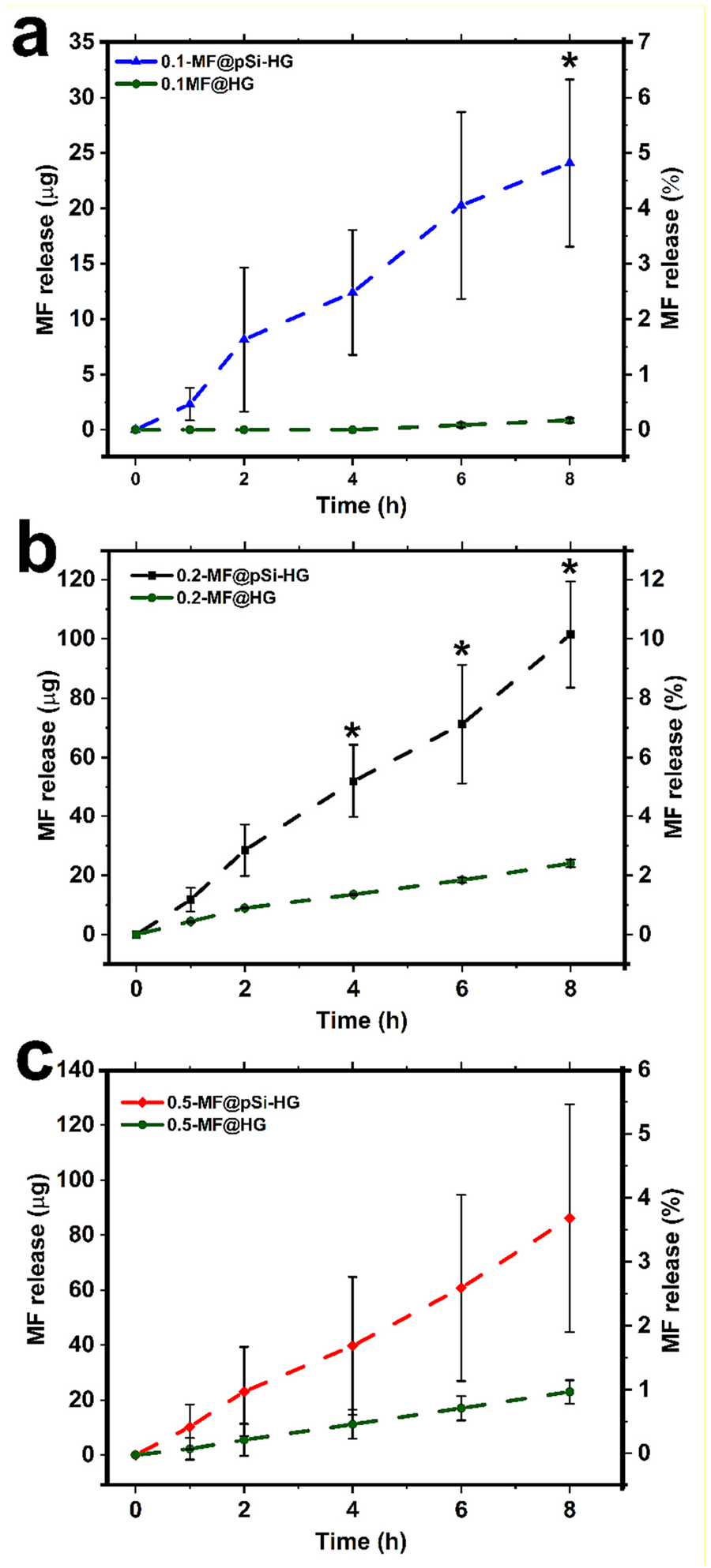
Temporal *in-vitro* release curves comparing cumulative release of mometasone furoate (MF) from hydrogel samples that contained either MF-loaded pSi particles or an equivalent mass of pure MF. Elution studies were performed at 34 °C, with the formulation contained in a sealed dialysis bag with simulated nasal fluid (SNF) in the receptor medium. The amount of hydrogel formulation in each dialysis bag was 500 μL. (a) cumulative MF release from 0.1-MF@HG (green) and 0.1-MF@pSi-HG (blue), (b) cumulative MF release from 0.2-MF@HG (green) and 0.2-MF@pSi-HG (black), and (c) cumulative MF release from 0.5-MF@HG (green) and 0.5-MF@pSi-HG (red). Data for each MF release time point is presented as mean ± SD of three independent experiments and was analysed by *t*-test comparing the difference of means of corresponding composite hydrogels and controls (* = *P* < 0.05).

Quantification of the rate of elution of the mometasone furoate drug from the formulations can allow prediction of the steady-state concentration of drug in the tissue, an important parameter in achieving a therapeutic effect. Static elution measurements were performed in a one-compartment elution cell, where the hydrogel formulation was loaded into a dialysis tube, the tube was sealed, and then the assembly was placed in a vial containing a buffer solution. The elution cell was maintained at 34 °C, the buffer was sampled periodically, and the eluted MF was quantified by HPLC. Because the amount of MF in the pSi particles was fixed at 28 *wt*%, the total amount of MF in each formulation was adjusted by changing the concentration of pSi particles in each hydrogel, as described above. The three different MF@pSi-HG formulations containing 0.1, 0.2, and 0.5 *wt*% MF (*i.e.,* 0.1-MF@pSi-HG, 0.2-MF@pSi-HG, 0.5-MF@pSi-HG) and their corresponding controls, consisting of free MF loaded into plain hydrogel (*i.e*., 0.1-MF@HG, 0.2-MF@HG and 0.5-MF@HG), were subjected to the elution protocol using two different buffers as receptor media: simulated nasal fluid (SNF) and a 50:50 (*v*/*v*) mixture of SNF with ethanol. Cumulative temporal MF release curves for SNF, reported as total mass and percentage of MF released from each formulation, are presented in Fig. 5. The pSi-based formulations consistently showed a higher rate of elution of MF relative to the free MF formulations. Such enhanced rates of elution are characteristic of nano porous drug carriers, and this phenomenon is generally attributed to the ability of the nanostructure to suppress drug crystallization [55] and to expose a large surface area of drug to the aqueous elution medium [56].

In order to better simulate the sink condition of the physiological milieu, release of MF from the hydrogel formulations was also studied in a 50:50 (*v*: *v*) mixture of SNF and ethanol. A significant increase in the amount of drug released was observed with the mixed solvent system (Fig. S4, Supporting Information). Whereas between 3 and 10% (depending on formulation) of MF was released into SNF in the first 8 h, 20–40% of the MF payload was released into the mixed aqueous-ethanol solvent in this same time period. This can be explained by the lipophilic nature of the corticosteroid; inclusion of ethanol in the elution medium helps to solubilise MF. The use of ethanol in the receptor medium is in accordance with the literature [57,58]. It is demonstrated that the addition of ethanol as co-solvents to ensure sink conditions does not alter the structure and barrier function of the skin [59]. The SNF:ethanol (50:50) mixture in the receptor phase assures perfect sink conditions are maintained, which has been previously shown and is attributed to considerably high solubility of MF in ethanol (>1.5 mg/mL)MF [60]. Thus, when SNF was used as receptor medium the amount of MF released from the free MF formulations 0.1-MF@HG, 0.2-MF@HG and 0.5-MF@HG over the 8 h measurement period was between 0.2 and 2.4% (Fig. 5), whereas this rose to between 0.7 and 8% when 50:50 SNF: ethanol was employed (Fig. S4, Supporting Information). A similar trend was observed with the control formulations.

### 3.5. Ex-vivo evaluation of toxicity of MF@pSi-HG formulations on human nasal tissue

**Fig. 6.**
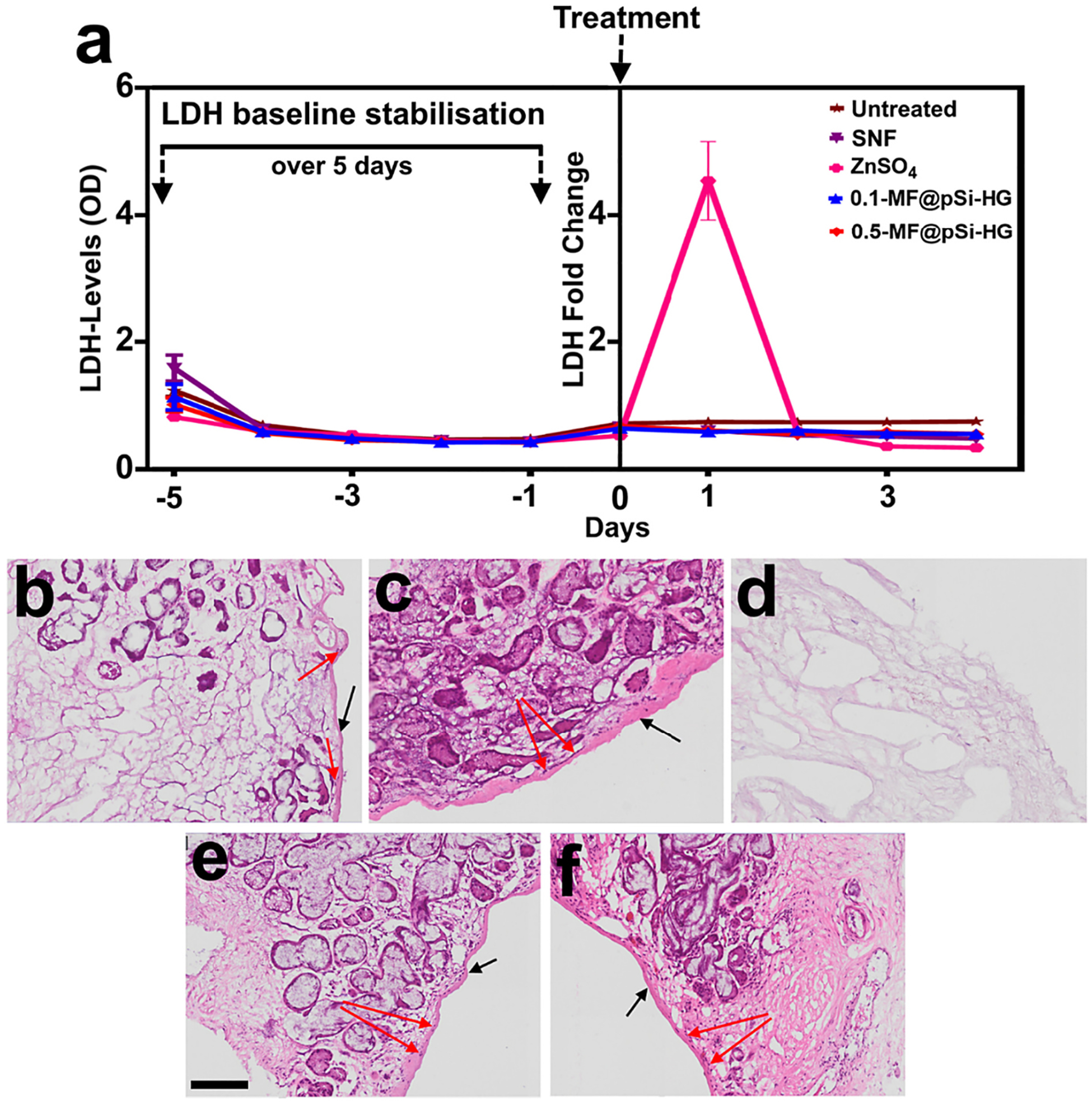
(a) Lactate dehydrogenase (LDH) assay results evaluating toxicity of formulations applied to human nasal mucosal tissue, measured 5 days pre-exposure and 5 days post-exposure (including day 0). After exposure, tissues treated with hydrogel formulations containing MF-loaded pSi particles (0.1-MF@pSi-HG and 0.5-MF@pSi-HG) and SNF and SNF control solution were found to maintain LDH at baseline levels, while 1% *w*/*v* of ZnSO_4_ in DMEM (positive control) elicited a significant cytotoxic and severe inflammatory response. All the data is presented as mean ± standard deviation of three repeats. Histology images of nasal mucosa tissue at 10× magnification (b) treated with 0.1-MF@pSi-HG, (c) treated with 0.5-MF@pSi-HG, (d) treated with 1% *w*/*v* ZnSO_4_, (e) tissue treated with SNF, (f) untreated tissue. The scale bar is 100 μm and same for all histology images. The black arrows mark the olfactory epithelium and red arrows point to basal cells. All the tested control and test samples except for the 1 *w*/*v* % ZnSO_4_ were nontoxic with comprehensive obliteration of the olfactory epithelium and basal cell layer.

The toxicity of the hydrogel formulations that incorporated MF-loaded pSi particles was systematically evaluated in human nasal tissue sections. Tissue samples were collected from informed and consenting patients undergoing surgery for nasal obstruction at the Greenslopes Hospital, Brisbane, Australia. The toxicity assay involved quantification of lactate dehydrogenase (LDH) released from the nasal tissue sections in response to the treatments. LDH is released by stressed and dying cells, and it has been employed as an effective biomarker of cellular toxicity [61]. In the present study, LDH levels in the human nasal tissues were monitored in growth media for five days. In this period, the LDHs level were observed to decrease initially and then reach stable baseline levels (Fig. 6a, pre-treatment). The tissues (*n* = 3 per set) were then treated with test or control formulations, and LDH levels were monitored for an additional 5 days. One control set of human nasal tissue sections were left untreated. A second control set of tissue samples were treated with SNF (negative control). A third set of tissue samples were administered 1% *w*/*v* ZnSO_4_ (made in DMEM) solution as a positive control. Zn (II) at this dose is reported to elicit strong toxic effects on nasal mucosal tissue [62]. Only this Zn (II) positive control resulted in a spike in LDH levels (Fig. 6a, post-treatment). Two sets of nasal tissue sections were treated with one of the MF@pSi-HG formulations (0.1 and 0.5 *wt*%). These did not show any detectable change in LDH levels, similar to the untreated and SNF-treated (*i.e.,* negative control) tissue sections.

The results from the LDH assay were confirmed by histological analysis (H&E staining). As depicted in Fig. 6b–c and Fig. 6e–f, the olfactory epithelium lining and basal cells remained intact for the untreated, SNF treated,0.1-MF@pSi-SG and 0.5-MF@pSi-SG. As expected, post-treatment with 1% *w*/*v* ZnSO_4_, there were clear and widespread changes characterised by degeneration of basal cells and severe inflammatory cell infiltration (Fig. 6d). In agreement with the LDH assay data, both the pSi-HG composite formulations (0.1-MF@pSi-HG and 0.5-MF@pSi-HG) did not affect nasal mucosal tissue morphology and integrity. It is worth mentioning, that the nasal mucosal tissues toxicity data shows that even the highest drug content formulations (with 0.5 wt % equivalent amount of drug) which also contain the highest amount of porous silicon particles (13 mg of MF loaded particles/g of gel) were non-toxic. While the formulation with the lowest drug and porous silicon particles did not show any toxicity. This provides us with confidence that other formulations with drug and particle concentration within this range will impose non-significant toxicity challenge. Therefore, 0.2-MF@pSi-HG formulation was not assessed for toxicity with human tissue.

### 3.6. MF deposition on human nasal tissue from pSi-HG and HG formulation

**Fig. 7.**
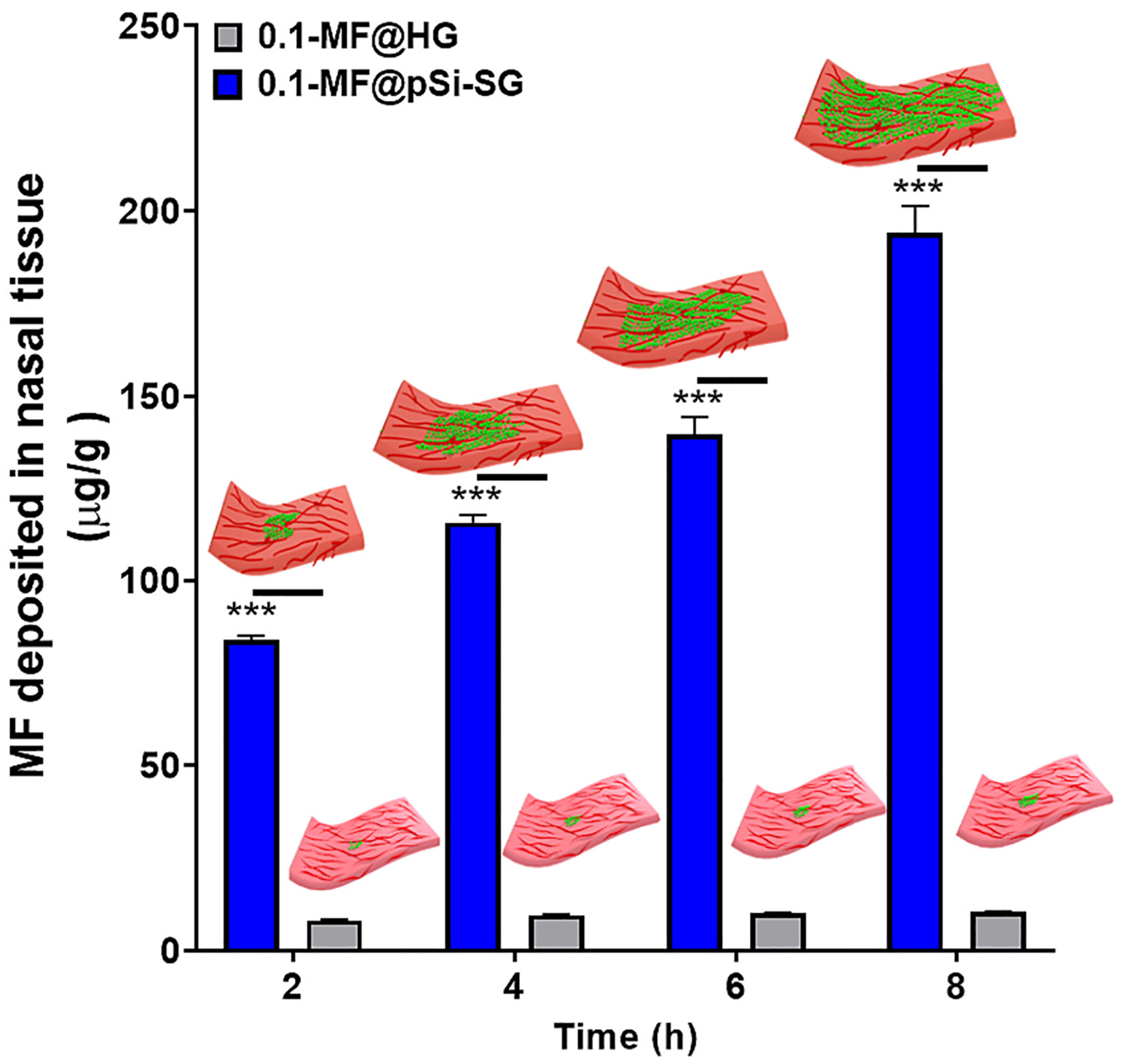
Infiltration of mometasone furoate (MF) into human nasal mucosal tissue from pSi microparticle-containing hydrogels (0.1-MF@pSi-HG) and from control hydrogels containing free MF (0.1-MF@HG). Deposition of MF is quantified per gram of nasal tissue, measured 2 h, 4 h, 6 h, and 8 h after application of the relevant formulation. Both formulation types contain the same mass of MF. A gradual increase in the amount of drug deposited with time is observed for the 0.1-MF@pSi-HG formulation, while the 0.1-MF@HG control deposits a substantially lower amount (almost 19-times less) of drug per gram of nasal tissue over the 8 h span of the experiment. Data at each time point is presented as mean ± SD of three independent experiments and *t*-test indicated that difference of means of 0.1MF@pSi-HG and 0.1MF@HG at each time point was statistically significant (*** *P* < 0.0005).

As the pSi-HG composite formulations displayed satisfactory rheomechanical characteristics, *in-vitro* drug release performance, and toxicity profiles, we next quantified drug deposition into human nasal tissues, in order to evaluate the potential suitability of the materials for delivery of therapeutically relevant drug doses. As most common MF formulations of in the market contains 0.1 *wt*% drug (*e.g*. Telnasal or Nasonex^®^ in Australia), we tested the 0.1-MF@pSi-HG formulation, which contained 0.1 *wt*% MF. The experiments were performed on human nasal mucosal tissue in a Franz cell maintained at 34 ± 1 °C for periods of between 2 and 8 h. In a typical experiment, the dorsal side of the nasal tissue was exposed to 0.5 mL of the 0.1-MF@pSi-HG formulation, and the tissue was removed at a given time point to quantify drug uptake. The amount of drug deposited accumulated in the tissues increased steadily as a function of time (Fig. 7); Control 0.1-MF@HG formulations only deposited 10.4 ± 0.1 μg/g of MF in the same time period after 8 h of exposure. Whereas the maximum amount of MF deposited from the 0.1-MF@pSi-HG formulation was 194 ± 7 μg per gram of human nasal tissue, measured by homogenization of the tissue after 8 h of exposure which is 19-fold higher compared to control MF@HG (Fig. 7). The drug deposition results demonstrate the advantage of incorporating MF@pSi particles into the HG: inclusion of the pSi nanostructure in the hydrogel formulation increased MF deposition in human nasal tissue almost 19-fold in an 8-h time period. The significantly higher amount of MF deposited in the tissues is attributed to the high loading capacity of the pSi particles, faster release rate and to their ability to maintain MF in a more bioavailable amorphous form [63].

## 4. Conclusions

This work established that mesoporous silicon-based microparticles can substantially increase both the rate of deposition and the quantity of steroid drug (mometasone furoate, MF) that can be deposited into nasal tissues using a thermoresponsive hydrogel, relative to a control involving only the free drug in the same poloxamer-based hydrogel. The addition of the MF-loaded pSi particles at concentrations up to 1.3% by mass did not adversely impact the rheological properties of the thermoresponsive hydrogel. *In-vitro* experiments established that the MF-loaded pSi particles increased the rate of MF release from the hydrogel host, presumably by inhibiting crystallization of the drug and thus enhancing the kinetics of drug dissolution. This increased rate of release was validated in two types of media: simulated nasal fluid (SNF) and SNF containing ethanol (which is a better solvent for the poorly water-soluble MF). The designed HG formulations displayed no acute toxicity toward human nasal mucosal tissue. Experiments using human nasal tissue in a Franz cell maintained in physiologic conditions showed an almost 19-fold increase in the amount of drug that could be deposited from the pSi-hydrogel composite formulation in an 8-h time period, compared with free (microcrystalline) MF suspended in the same poloxamer hydrogel host. Together, these results indicate that a mesoporous Si drug carrier can enable improved performance for intranasal delivery of hydrophobic drugs such as steroids.

## Supplementary Material

Supporting information

## Figures and Tables

**Table 1:** Composition of the MF-loaded hydrogel (MF@HG) and MF-loaded porous Si particle-hydrogel composite (MF@pSi-HG) formulations used in this work.

Formulation	MF (*wt*%)	MF required (mg)/g of HG	MF@pSi added (mg)/g of HG	pSi (mg)/g of HG
0.1-MF@HG	0.1	1	0	0
0.2-MF@HG	0.2	2	0	0
0.5-MF@HG	0.5	5	0	0
0.1-MF@pSi-HG	0.1	1	3.6	2.6
0.2-MF@pSi-HG	0.2	2	7.2	5.2
0.5-MF@pSi-HG	0.5	5	18	13

Note: The amount of MF@pSi incorporated is based on 28 wt% loading of MF in pSi (*e.g*. 28 mg of MF in 100 mg of MF@pSi formulation), which was achieved by the vacuum loading process described in Section 2.3.

## Data Availability

Data will be made available on request.
