# Supplementary material for: Porous silicon embedded in a thermoresponsive hydrogel for intranasal delivery of lipophilic drugs to treat rhinosinusitis
Source: J Control Release. Author manuscript; Available in PMC 2024 Oct 17. (PMC11484479; doi:10.1016/j.jconrel.2023.09.045)
Supplement: Supporting information [file NIHMS2028867-supplement-Supporting_information.pdf]

Supplementary information

Porous Silicon Embedded in a Thermoresponsive Hydrogel for Intranasal Delivery of Lipophilic Drugs

Shrishty Bakshi[1§], Preeti Pandey[1§], Yousuf Mohammed[2], Joanna Wang[3], Michael J. Sailor[4], Amirali Popat[1*], Harendra S Parekh[1,*] Tushar Kumeria[1, 5, 6 *]

[1] School of Pharmacy, The University of Queensland, Queensland 4102, Australia
[2] Therapeutics Research Group, Diamantina Institute, University of Queensland, Brisbane, Queensland 4102, Australia
[3] Department of Radiology, School of Medicine, Stanford University, Stanford, California 94305, United States of America
[4] Department of Chemistry and Biochemistry, University of California-San Diego, La Jolla, California 92093, United States of America
[5] School of Materials Science and Engineering, The University of New South Wales, New South Wales 2052, Australia
[6] Australian Centre for Nanomedicine, The University of New South Wales, New South Wales 2052, Australia

Section S1. Rheological assessment of porous silicon hydrogel formulations

Table S1. A summary of key rheological properties of pSi incorporated hydrogel formulations.

**Formulation**	**Gelation temperature (°C)**	**Viscosity at gelation temperature (Pa.s)**	**Tan (δ) at gelation temperature**
**Blank HG**	27.4	0.18	0.62
**0.1-MF@pSi-HG**	27.9	0.18	0.40
**0.2-MF@pSi-HG**	28.3	0.16	0.13
**0.5-MF@pSi-HG**	28.5	0.20	0.78

Note: All the numbers are an average of three individual measurements.

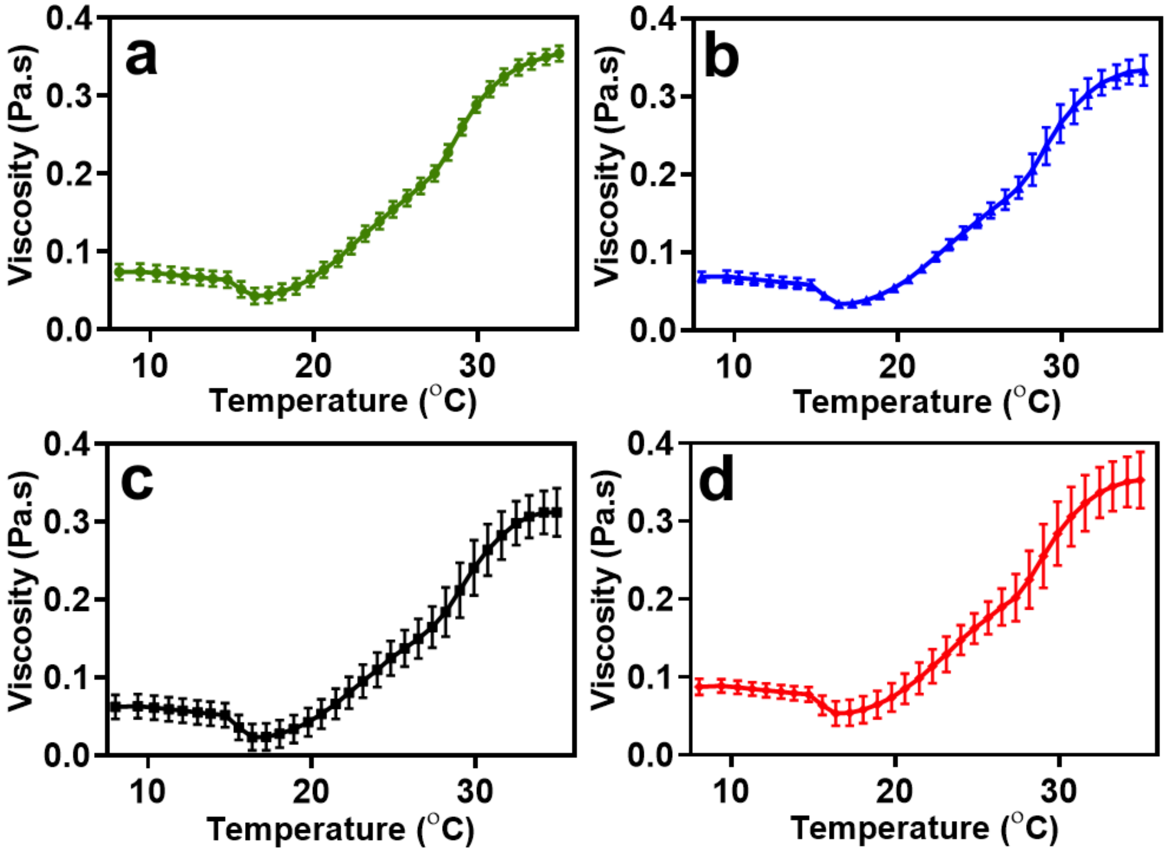

Figure S1. Temperature dependent viscosity data for (a) blank HG, (b) 0.1-MF@pSi-HG, (c) 0.2-MF@pSi-HG, and (d) 0.5-MF@pSi-HG formulations. Error bars represent ± SD.

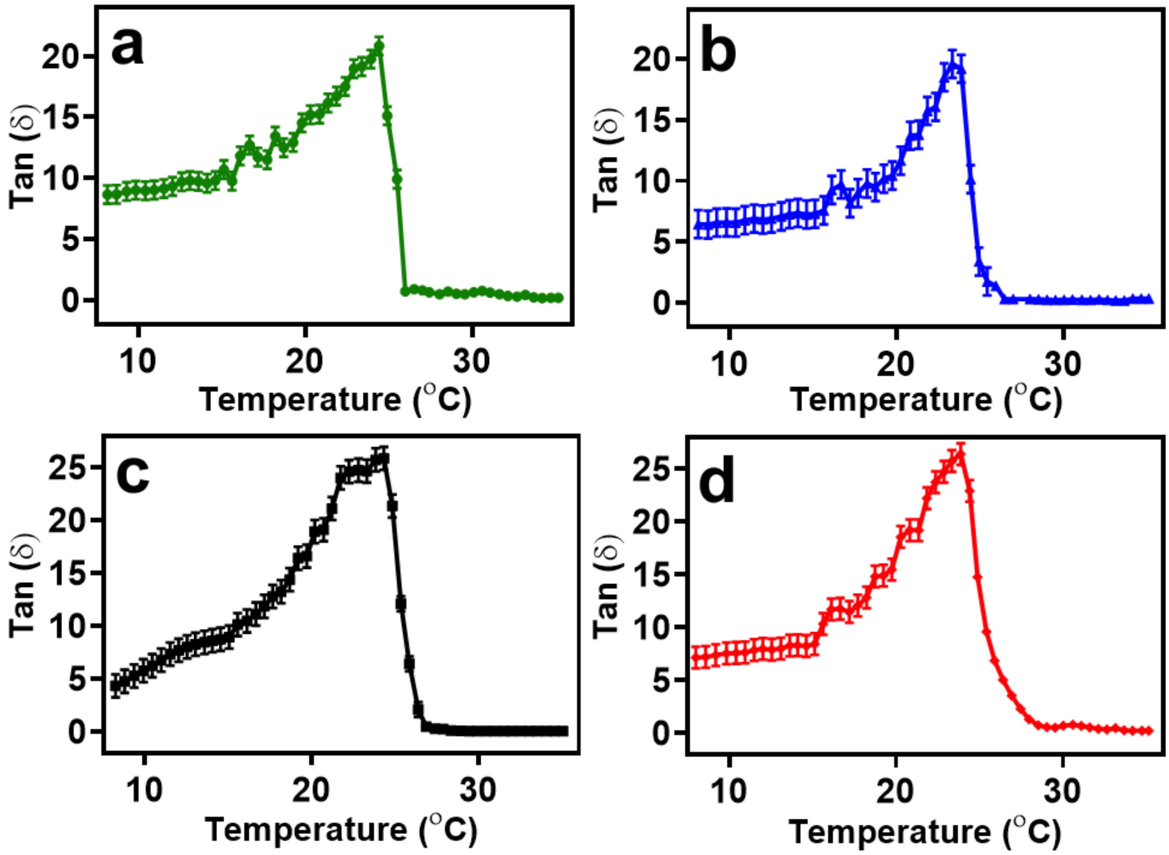

Figure S2. Temperature dependent tan delta (δ) data for (a) blank HG, (b) 0.1-MF@pSi-HG, (c) 0.2-MF@pSi-HG, and (d) 0.5-MF@pSi-HG formulations. Tan delta values less than 1 at the gelation temperature in all four cases indicates good quality of the gels. Error bars represent ± SD.

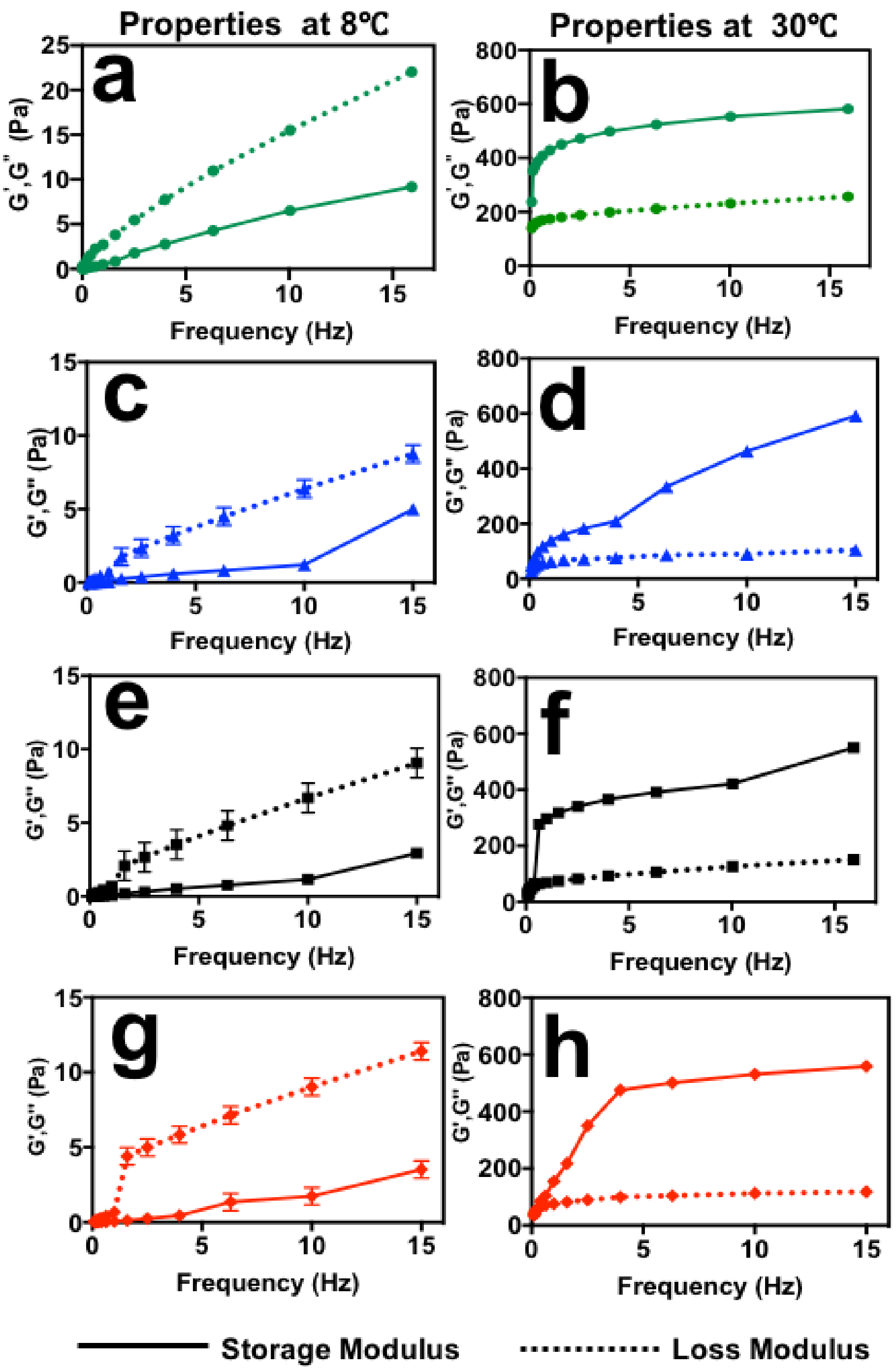

Figure S3. Frequency-dependent changes in viscoelastic properties of selected formulations at 8°C and 30 °C (a & b) blank hydrogel, (c & d) formulation 0.1-MF@pSi-HG, (e & f) formulation 0.2-MF@pSi-HG, (g & h) formulation 0.5-MF@pSi-HG. At the temperature above the gelation temperature (> 30°C), the storage modulus is higher than the loss modulus in all the formulations, indicating that changes in frequency did not influence the gel structure. (n = 3; error bars represent ± SD)

Section S2. *In-vitro* drug release in 50:50 mixture of SNF and ethanol

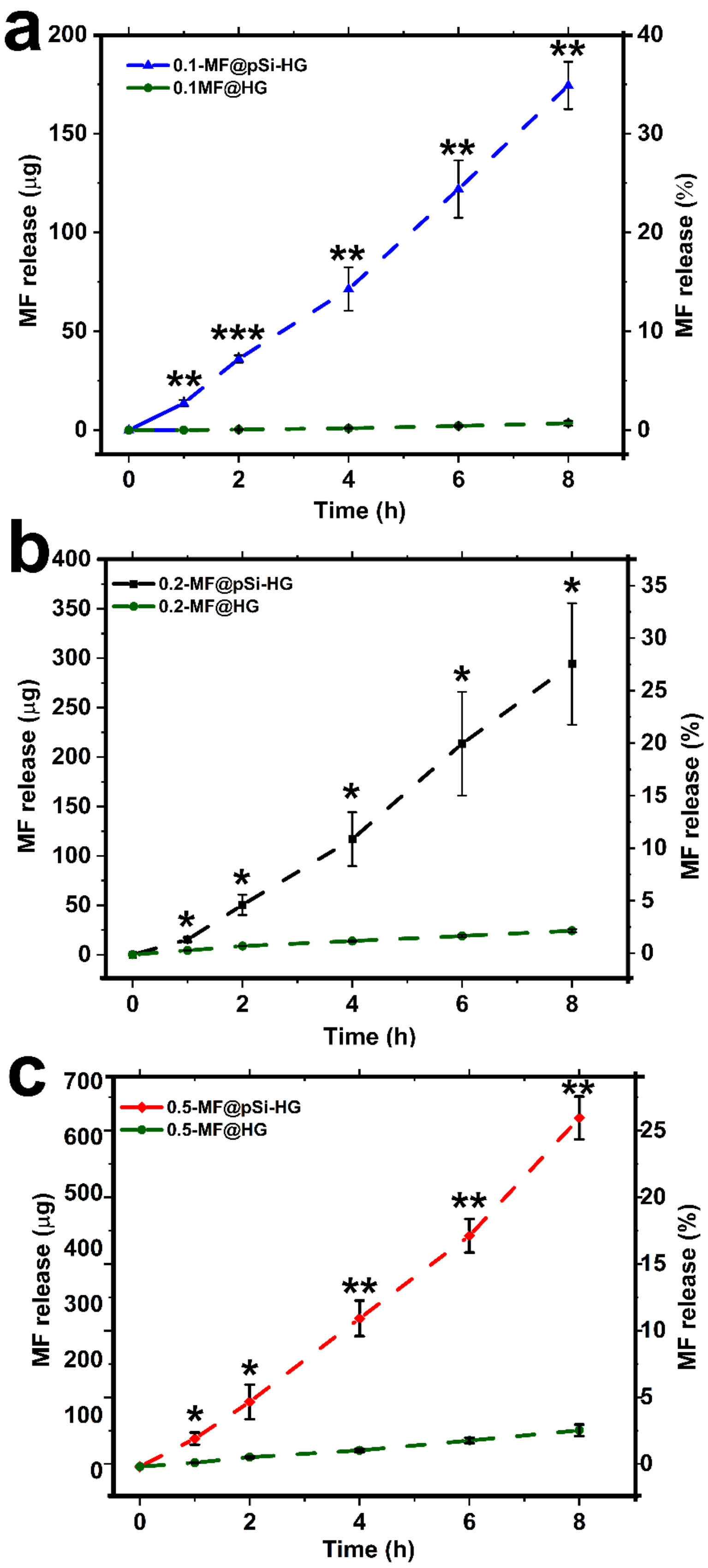

Figure S4. Cumulative mometasone from pSi-HG composite and corresponding MF@HG formulations (green traces) conducted at 34 °C in a dialysis bag, using a 50:50 mixture of

simulated nasal fluid (SNF) and ethanol (EtOH) in the receptor medium. The amount of composite hydrogel applied in each was kept constant at 500 µL. (a) cumulative µg MF release from 0.1-MF@HG (green trace) and 0.1-MF@pSi-HG, cumulative % MF release from 0.1-MF@HG (green trace) and 0.1-MF@pSi-HG, (b) cumulative µg MF release from 0.2-MF@HG (green trace) and 0.2-MF@pSi-HG, cumulative % MF release from 0.2-MF@HG (green trace) and 0.2-MF@pSi-HG, (c) cumulative µg MF release from 0.5-MF@HG (green trace) and 0.5-MF@pSi-HG, (f) cumulative % MF release from 0.5-MF@HG (green trace) and 0.5-MF@pSi-HG. Data at each time point is presented as mean ± SD of three independent MF release experiments and was analysed by t-test comparing the difference of means of corresponding composite hydrogels and controls (* = $P < 0.05$, ** = $P < 0.001$, and *** = $P < 0.0005$).
